# In Vitro Studies on 3D-Printed PLA/HA/GNP Structures for Bone Tissue Regeneration

**DOI:** 10.3390/biomimetics9010055

**Published:** 2024-01-19

**Authors:** Andreea-Mariana Negrescu, Aura-Cătălina Mocanu, Florin Miculescu, Valentina Mitran, Andreea-Elena Constantinescu, Anisoara Cimpean

**Affiliations:** 1 Department of Biochemistry and Molecular Biology, Faculty of Biology, University of Bucharest, 91-95 Splaiul Independentei, 050095 Bucharest, Romania; andreea.ne94@gmail.com (A.-M.N.); valentina.mitran@bio.unibuc.ro (V.M.); 2 Department of Metallic Materials Science, Physical Metallurgy, National University of Science and Technology Politehnica Bucharest, 313 Splaiul Independentei, J Building, District 6, 060042 Bucharest, Romania; mcn_aura@hotmail.com (A.-C.M.); f_miculescu@yahoo.com (F.M.); andreeaelena01c@gmail.com (A.-E.C.)

**Keywords:** bone tissue regeneration, 3D printing, PLA-HA matrix, graphene, osteogenic differentiation, immune response

## Abstract

The successful regeneration of large-size bone defects remains one of the most critical challenges faced in orthopaedics. Recently, 3D printing technology has been widely used to fabricate reliable, reproducible and economically affordable scaffolds with specifically designed shapes and porosity, capable of providing sufficient biomimetic cues for a desired cellular behaviour. Natural or synthetic polymers reinforced with active bioceramics and/or graphene derivatives have demonstrated adequate mechanical properties and a proper cellular response, attracting the attention of researchers in the bone regeneration field. In the present work, 3D-printed graphene nanoplatelet (GNP)-reinforced polylactic acid (PLA)/hydroxyapatite (HA) composite scaffolds were fabricated using the fused deposition modelling (FDM) technique. The in vitro response of the MC3T3-E1 pre-osteoblasts and RAW 264.7 macrophages revealed that these newly designed scaffolds exhibited various survival rates and a sustained proliferation. Moreover, as expected, the addition of HA into the PLA matrix contributed to mimicking a bone extracellular matrix, leading to positive effects on the pre-osteoblast osteogenic differentiation. In addition, a limited inflammatory response was also observed. Overall, the results suggest the great potential of the newly developed 3D-printed composite materials as suitable candidates for bone tissue engineering applications.

## 1. Introduction

In today’s society, bone defects caused by trauma, tumour resections, infections, autoimmune disorders and congenital anomalies present a significant clinical problem, affecting both the patient and the healthcare services [1]. Modern treatment options for these bone defects consist of bone grafting (e.g., autographs, allografts, xenografts) and conventional metallic implants. However, these methods present major drawbacks in terms of high surgery costs, donor site pain, limited bone availability, inflammation, stress shielding effect, secondary removal surgery and a prolonged recovery time [2]. To solve this issue and overcome the limitations imposed by the traditional approaches, an alternative strategy for bone tissue repair has emerged in the form of bone tissue engineering (BTE), an active on-going developing field that combines bone cells with artificial templates known as scaffolds in order to assemble a structure capable of restoring and maintaining the inherent architecture and function of the damaged bone tissue [3,4]. Scaffolds are supporting structures made either from polymers (natural and/or synthetic) or, in some cases, bioactive ceramics, which act as biomimetic extracellular matrices (ECMs) for bone cells [5]. For this reason, the scaffolds must be designed accordingly to mimic the natural architecture of the ECM, thus providing the cells with a variety of physical, chemical, and biological cues which helps stimulate cell adhesion [6]. Moreover, a properly designed scaffold should go even further and facilitate cell spreading, migration, proliferation and differentiation in order to encourage host tissue integration and new bone formation in vivo [3]. Last but not least, scaffolds should be biocompatible, i.e., non-immunogenic, non-thrombogenic and resistant to infections [7]. Based on the above, an appropriate designed scaffold should be capable of (i) maintaining the original shape and integrity of the bone tissue (excellent mechanical strength which can withstand external pressure); (ii) promoting cell adhesion and growth (favourable biocompatibility); and (iii) degrading in an appropriate amount of time which allows the bone tissue enough time to completely heal and regenerate [8]. 

In the last couple of years, various technologies such as electrospinning, particulate leaching, solvent casting and laser ablation have been used in order to design and fabricate biodegradable scaffolds [9,10]. However, the aforementioned techniques can still pose some limitations in the form of poor reproducibility/repeatability and a limited ability to control essential surface architectural features such as pore size, shape, distribution and interconnectivity [1]. In tissue engineering, especially for bone tissue regeneration, such surface characteristics are critical. Therefore, the newly fabricated scaffolds must be precisely designed in order to provide the cells with a proper microenvironment that meets the need of new bone ingrowth [11]. Recently, additive manufacturing techniques or 3D printing (denoted in standard definition terms according to the ISO/ASTM 52900-2021 standard [12], currently in use) technologies emerged as a solution, allowing for the production of precise and complex 3D interconnected porous scaffolds in a reproducible manner, with almost no human intervention, through the deposition of material using the print head of a nozzle. Based on a desired 3D model, it locates and assembles the selected biomaterials to fabricate various biomedical devices such as artificially implanted stents, tissues and organs [13,14]. Moreover, the 3D printing technology does not only possess an excellent control over the architecture and microstructure of the scaffold but also allows growth factors and living cells to be integrated into it during the manufacturing process [8]. This feature enables the fabrication of custom-made scaffolds for an individual treatment that meets the clinical needs of the patient [15]. 

In the present study, the 3D-printed composite scaffolds were fabricated using one of the 3D printing technologies, fused deposition modelling (FDM), which requires first the material extrusion (also recently known as MEX in the current standard [12]) in the form of filaments to be afterwards used as feedstock (bulk raw material supplied for the building process). This enables thermoplastic biomaterials that not only possess a favourable mechanical strength but are also biocompatible to be 3D printed [16]. In this regard, a wide range of biomaterials such as natural/synthetic polymers and bioceramics fit into the technical specifications of the FDM technology and are commonly used for tissue engineering scaffolding. Nevertheless, despite the extensive range of usable biomaterials, their selection is just as important as the scaffold’s surface architecture and can directly influence the conditions that lead to a successful therapeutic outcome [17]. With this in mind, in our previous work [18] two categories of precursor materials ((i) prime materials—acrylonitrile butadiene styrene (ABS) and polylactic acid (PLA); and (ii) reinforcement materials—natural hydroxyapatite (HA) and graphene nanoplatelets (GNPs) of different dimensions (grade_M and grade_C)) were comprehensively investigated in terms of their physico-chemical, morphological and in vitro biological behaviour, and the obtained results led to the successful selection of materials with adequate features for the envisioned biomedical applications. Thus, the development of the composite filaments was carried out using PLA as a polymeric matrix with both GNP_grade M and natural HA as reinforcement materials. From data reported in the literature, PLA proved to be a suitable candidate for tissue engineering manufacturing due to its low immunogenicity, its batch variation and the undemanding modulation of its biochemical and physical characteristics by blending it with different nanofillers [19]. Moreover, PLA-based scaffolds are self-standing structures with the ability to promote cell attachment, anchorage, proliferation and differentiation [20]. In addition, bare PLA is fully resorbable and has a very slow degradation rate. Such features turn PLA scaffolds into suitable candidates for planned reabsorption and are therefore the preferred base materials for artificial tissue/scaffold manufacturing for bone tissue regeneration [21]. However, in spite of the multiple advantages, PLA-based scaffolds do not possess osteoinductive and osteoconductive properties, thus being considered incapable of supporting the complete regeneration of the bone defect on their own [22]. With this in mind, the addition of mineral-based biomaterials into the PLA matrix could represent a viable strategy in improving the osteo-promotive ability of the 3D-printed scaffolds. Hydroxyapatite, one of the most extensively studied biomaterials mainly due to its similarity to the mineral phase of natural human bone, is capable of promoting bone cell differentiation and increasing the deposition of the newly formed tissue at the site of implantation [23]. Furthermore, recent studies have shown that by combining a synthetic polymer with ceramic material, the resulting composite scaffold is capable of improving the osseointegration rate and new bone tissue formation when compared to the simple form of each biomaterial [21,24]. 

In the current work, novel 3D-printed products based on PLA/HA/GNP precursors, with tuneable chemical, physical and mechanical properties, have been fabricated, and their in vitro behaviour has been investigated. Moreover, due to the poor mechanical properties (e.g., low fracture toughness and tensile strength) of the PLA-HA scaffold, graphene-based materials have been added directly into the ceramic matrix and integrated into the polymeric scaffold without any binding materials or chemically routed methods. The reason why graphene has been chosen as a reinforcement material is mainly down to its unique structure and its exceptional mechanical and electrical properties, features which have transformed graphene into a rapidly emerging biomaterial with biomedical applications [25]. Moreover, graphene-based biomaterials have been proven to improve cell adhesion, growth, proliferation and differentiation [26]. Considering the essential biocompatible character of the implantable products and the well-known toxicity and effect upon the DNA structure of another nanomaterials (such as carbon nanotubes), they were excluded based on the literature reports, regardless of their potential to improve some of the printable materials properties related to the FDM technique [27,28,29,30].

The prospected novel vision outlined in this study addressed, for the first time, the in vitro behaviour corresponding to the tuning parameters by which the developed PLA/HA/GNP-based scaffolds were manufactured. The full design of the extensive experimental programme envisioned for this subject targeted the modulation of several essential parameters for the composite filaments’ development: the type of polymeric matrix, the type of GNP materials, the HA ratio and particles size range, and finally, the GNP ratio. Given that a full factorial design would have proved disadvantageous due to the exponential increment of sample number requirements with the number of factors/inputs/parameters to be considered, we opted for an alternative strategy—fractional factorial designs—which allowed for the comparative assessment of a maximum of two parameters in turn. This led also to the optimisation of sample numbers before moving forward to investigate another set of two parameters (reduced number of samples, implicitly reduced fabrication costs and unnecessary use of precursor materials). The incipient study regarding the influence of adding HA into the PLA matrix, at different ratios (0–50 wt.%) and with different particles size ranges, can be found at reference [31]. The outcome revealed only one optimum range for the particles size, regardless of the input ratio. Afterwards, as mentioned above, we tested, from each category of precursor materials, two types according to the literature, and the results were reported in reference [18]. In the final step, after excluding all deficient or biologically inappropriate routes, we evaluated the influence of the GNP addition in modulated ratios (0–5 wt.%) combined with the already established parameters for the HA component (ratio of 0–50 wt.%), and the results are under consideration for publication elsewhere.

Based on the abovementioned logical flow and experimental outcomes that facilitated the elimination of the 40–50 wt.% HA and 4–5 wt.% GNP (as to avoid nozzle clogging and fragile filaments input), herein, the programmed in vitro investigations targeted only composite materials with printable features developed at the optimised HA/GNP ratios in the maximum range of 0–30 wt.%/0–3 wt.%. The evaluation was performed in close regard to effective cost and time management for the fabrication of samples, which takes into account also the necessary identical samples/sample type/in vitro test in order for the replication principle to work.

## 2. Materials and Methods

### 2.1. Samples Preparation

Some initial precursor materials—*PLA* in form of granules of a natural colour, which were 2 ± 0.05 mm in diameters (Merck KGaA, Darmstadt, Germany), and x*GNP*^®^, grade M, in the form of plates ~7 nm in thickness and 25 μm in diameter (graphene nanoplatelets, XG Sciences Inc., Lansing, MI, USA) —were acquired from local vendors. Precursors were used for the preparation of composite materials without any chemical or physical treatment. A complex description of the precursor materials is provided in reference [18]. The bioceramic material (HA) was synthesised from bovine bones through an already established, sustainable and reproducible conversion procedure (based on successive thermal treatments), as previously reported [32,33]. After ball mill grounding (450 rpm/2 h) and granulometric sorting (Retsch GmbH, Haan, Germany), only the optimal dimensional sort of the resulting HA powder (<40 μm) was chosen for this study, based on our previous findings [31].

The fabrication of the composite materials involved the incorporation of different ratios of HA (in the 0–30 wt.% range; 10 wt.% increment) and GNP (in the 0–3 wt.% range; 1 wt.% increment) materials into the PLA matrix. The optimal ranges for the HA/GNP ratio were established in our previous work—if the incorporated amounts exceed these values, the mechanical uptake decreases significantly, while the rigidity/stiffness degree indicates an opposite trend [18]. Further, the process required (i) the mechanical homogenisation of all precursor materials (50 rpm/1 h) in a tumbler mixer (Inversina, Bioengineering AG, Zürich, Switzerland) and (ii) thermal homogenisation (190 °C), via continuous stirring on the magnetic stirrer hob, of the resulting mixtures. The denomination of the samples was set according to the function of the HA (e.g., 20% HA) and GNP (e.g., 2% GNP) ratios.

The obtained slurries were left to cure and harden and converted afterwards into small strips to be further used for the materials extrusion process (MEX) in form of filaments. Hence, only one MEX cycle performed at 10–15 rpm/200 °C/195 °C (rotational speed/barrel temperature/nozzle temperature) (Pro Filament Extruder, Noztek, West Sussex, UK) was targeted for the development of composite filaments with a uniform distribution of HA/GNP materials into the polymeric matrix. The extruded filaments (1.75 mm in diameter) were immediately air cooled and coiled on a spool.

The 3D printing of the composite products was performed via the FDM (FFF) technique on a 3D printer (WASP 2040 Turbo 2, Massa Lombarda, Italy) at an average printing temperature of 210 °C ± 5–20 °C function of the HA/GNP ratio (in order to avoid nozzle clogging), using the as-obtained composite filaments as feedstock. The 3D products were printed at an average speed of 10–25 cm/s on a glass support and a material flow of 90–110%. Although scaffold structures (with modulated spatial orientation of the printed lines) were developed with the composite filaments, here, for a proper in vitro cellular response, we deliberately designed the printing geometry as fused line-by-line with the plane surface and of a square shape with a side of 2 cm (similar to a cell testing plate) and an overlay of two printed layers (orientation of 90°).

The entire procedure for the scaffold manufacturing technology is provided in Figure S1 (Supplementary Material).

### 2.2. Scaffolds Characterisation

#### Morphological Evaluation

The morphological characteristics of the 3D-printed products were evaluated via scanning electron microscopy (ESEM Quattro microscope, Thermo Fischer Scientific™, Hillsboro, OR, USA) coupled with an auxiliary microanalysis EDS system (Thermo Scientific™ Pathfinder). The micrographs were recorded on the outer surface of the printed products, at an acceleration voltage of 15 kV and 10 mm working distance, in 5 random areas.

### 2.3. In Vitro Cellular Response

#### 2.3.1. Cell Culture Models 

For the biological experiments, two cell lines, namely, the MC3T3-E1 mouse pre-osteoblasts subclone 4 and the murine macrophage-like RAW 264.7, were purchased from the American Type Culture Collection (ATCC, Manassas, VA, USA) and grown in Dulbecco’s Modified Eagle Medium (DMEM, Sigma-Aldrich Co., St. louis, MO, USA) supplemented with 10% (*v*/*v*) foetal bovine serum (FBS, Life Technologies Corporation, Grand Island, NY, USA) and 1% (*v*/*v*) penicillin (10,000 units mL^−1^)/streptomycin (10 mg mL^−1^)/amphotericin B (25 µg mL^−1^) (Sigma-Aldrich Co., St. Louis, MO, USA) at 37 °C in a humidified atmosphere of 5% CO_2_. The medium was changed every 2–3 days, and when the culture reached about 80% confluence, the cells were trypsinised and seeded directly onto the surface of the analysed samples at initial densities that varied depending on the experimental approach. Hence, for cell viability/proliferation and morphology, the pre-osteoblasts were seeded at an initial density of 1 × 10^4^ cells/cm^2^, while the macrophages were seeded at 1.5 × 10^4^ cells/cm^2^. For the nitric oxide (NO) analysis and macrophage fusion investigation, the RAW 264.7 cells were seeded at an initial density of 8 × 10^4^ cells/cm^2^ and 5 × 10^3^ cells/cm^2^, respectively. Moreover, in order to assess the pre-osteoblast differentiation, an initial density of 4 × 10^4^ cells/cm^2^ was used. It is worth mentioning that the inflammatory response of the RAW 246.7 cells was evaluated under both standard and pro-inflammatory conditions (treatment with 100 ng mL^−1^ LPS from *Escherichia coli*), while the pre-osteoblast differentiation studies were conducted in the presence of an osteogenic medium containing specific factors such as ascorbic acid (50 µg mL^−1^), β-glycerophosphate (5 mM) and dexamethasone (10^−8^ M). For the inflammatory activity, the RAW 264.7 cells cultured on plastic in DMEM supplemented with 10% FBS and antibiotics/antimycotics without LPS were seen as the negative inflammatory control (TCPS (-)), while the macrophages grown on plastic in the presence of a pro-inflammatory agent (100 ng/mL LPS) were considered the positive control for inflammation (TCPS (+)). Similarly, for the osteogenic differentiation, the MC3T3-E1 cells were incubated in DMEM supplemented with 10% FBS and antibiotics/antimycotics without (TCPS (-)) and with osteogenic agents (TCPS (+)), and these two conditions were considered the negative and positive controls for differentiation, respectively. It is important to note that prior to osteoblast and macrophage seeding, the developed materials were sterilised via consecutive washes in 70% ethanol and Milli-Q water (30 min each), followed by a 30 min exposure on each side to an ultraviolet (UV) light in a vertical laminar flow cabinet.

#### 2.3.2. In Vitro Assessment of Cellular Survival

In order to investigate the potential cytotoxic effects of the analysed supports, in the first set of experiments, the cellular viability of the MC3T3-E1 cells was assessed through a qualitative method, namely, cell staining with the Live/Dead Viability/Cytotoxicity Kit (L-3224, Molecular Probes, Eugene, OR, USA) at 24 h and 96 h post seeding in accordance with the manufacturer’s instructions. For instance, at the end of the experimental time-points, the cellular monolayer was incubated for 10 min in the dark and at room temperature with 2 mM calcein-acetoxymethyl (AM) and 4 mM ethidium homodimer-1 (EthD-1), and the stained cells were visualised with an inverted fluorescence microscope (Olympus IX71, Olympus, Tokyo, Japan). The CellSense Dimension acquisition system Version 4.1. was used to capture representative images. Furthermore, the number of live osteoblasts and macrophages was quantified through the use of the Cell Counting Kit (CCK)-8 [34], and the cell viability was expressed as a percentage of the control sample represented by the standard tissue culture polystyrene (TCPS) substrate. 

#### 2.3.3. In Vitro Cellular Morphology Assessment 

To investigate the adhesion and morphological characteristics of the MC3T3-E1 and RAW 264.7 cells grown onto the surface of the 3D-printed composite supports, the fluorescent immunoreactive labelling of the actin cytoskeleton was performed. Thus, at the end of the experimental timepoints, the cellular monolayer was washed with phosphate-buffered saline (PBS) and fixed with 4% paraformaldehyde (PFA) for 20 min. Next, the cells were rinsed 3 times with PBS and the plasma membrane was permeabilised and blocked for non-specific binding with a solution of 0.1% Triton X-100/2% bovine serum albumin (BSA) for 30 min at room temperature. Following this step, the cells were washed again with PBS and incubated for 15 min at room temperature and in the dark, with AlexaFlour 488 phalloidin (20 µg mL^−1^, Invitrogen, Eugene, OR, USA). In the last step, the nuclei were stained with 4′,6-diaminodino-2-phenylindole (DAPI, Sigma-Aldrich Co., Steinheim, Germany) and the cellular monolayer was observed under an inverted fluorescent microscope (Olympus IX71, Olympus, Tokyo, Japan). Representative fields were captured using the CellSense Dimension system Version 4.1. 

#### 2.3.4. In Vitro Assessment of RAW 264.7 Immune Response

In order to observe the potential of the 3D-printed composite samples to influence and dictate the immune response, the production of NO, a pro-inflammatory marker, and the ability to induce the formation of foreign body giant cells (FBGCs) through macrophage fusion, were investigated. Quantification of NO production was conducted through a colorimetric assay, as previously reported [35]. Thus, for this test, 50 µL of culture supernatant and 50 µL of Griess reagent (Promega, Madison, WI, USA) were mixed and incubated for 10 min in the dark at room temperature. Subsequently, 50 µL of 0.1% *N*-1-napthylethylenediamine dihydrochloride aqueous solution were added to the mix, and the cells were incubated for another 10 min in the dark. Finally, the optical density (OD) of the product was measured at 550 nm using a microplate reader (FlexStation 3 microplate reader, Molecular Devices, San Jose, CA, USA), and a sodium nitrite standard curve was used to determine the nitrite concentration. The formation of FBGCs was investigated after 7 days of incubation, and the cellular monolayer was treated similar to the protocol in Section 2.3.3. Following the staining protocol, the cells were visualised with an inverted fluorescence microscope (Olympus IX71, Olympus, Tokyo, Japan), and the representative images were obtained using the CellSense Dimension acquiring system Version 4.1. Further on, the ImageJ operating system was used to determine the level of giant cell formation, which led to the establishment of the “multinuclearity index” meaning the percentage of nuclei found in the multinucleated cells (more than 3 nuclei) in ratio to the total number of nuclei found in the same field. 

#### 2.3.5. In Vitro Osteogenic Differentiation Assessment 

To determine whether the analysed samples were capable of supporting the osteogenic differentiation of MC3T3-E1 cells in osteoblasts, the alkaline phosphate (ALP) activity, collagen synthesis and extracellular matrix mineralisation were measured under osteogenic culture conditions. The intracellular ALP activity was determined at 7 and 14 days post seeding by means of the Alkaline Phosphatase Activity Colorimetric Assay Kit (BioVision, Milpitas, CA, USA), in compliance with the manufacturer’s instructions. Therefore, for this study, 80 µL of cellular lysate was mixed with 50 µL of 5 mM p-nitrophenylphosphate (pNPP), incubated at room temperature in the dark, and after 1 h, the reaction was interrupted by adding 20 µL of stop solution. The absorbance of the final product was measured at 405 nm with the help of a microplate reader (FlexStatio3 microplate reader, Molecular Devices, San Jose, CA, USA), and the concentration of the resulting product was determined by using a standard curve. Moreover, the protein concentrations were measured for each sample using the Bradford reaction, and the ALP activity was normalised to 1 µg of protein. The collagen synthesis was quantified after 14 and 28 days in culture with Sirius red staining, as previously described [36]. Briefly, the supports were rinsed with PBS and fixed with a solution of 10% PFA for 20 min. Afterwards, three washes with deionised water were performed, and the samples were maintained for 1 h in a 0.1% solution of Sirius red (Bio-Optica, Milano, Italy). At the end of the incubation time, the supports were air dried, and a solution of 0.2 M NaOH/methanol (1:1) was used to dissolve the staining. The OD was recorded at 540 nm with the help of a microplate reader (FlexStatio3, Molecular Devices, San Jose, CA, USA). The formation of extracellular calcium deposits was investigated at 4 weeks post seeding using Alizarin red staining. Briefly, after 4 weeks, the cellular monolayer was washed with PBS, fixed with a solution of 10% PFA for 20 min and incubated for 1 h with an Alizarin red solution. Afterwards, the dye was removed, and the cells were washed with distilled water. In order to evaluate the ECM mineralisation level, a solution of 5% perchloric acid was added in each well, and the OD of the resulting product was measured at 405 nm using a microplate reader (FlexStation3, Molecular Devices, San Jose, CA, USA).

#### 2.3.6. Statistical Analysis 

The statistical analysis of the obtained data was performed using the one-way ANOVA with Tukey’s multiple comparisons test (GraphPad Prism software Version 6, GraphPad, San Diego, CA, USA). The data are presented as the mean ± standard deviation (SD), and the *p* values below 0.05 are considered to be statistically significant. 

## 3. Results

### 3.1. Scaffolds Characterisation

#### Morphological Evaluation

**Figure 1:**
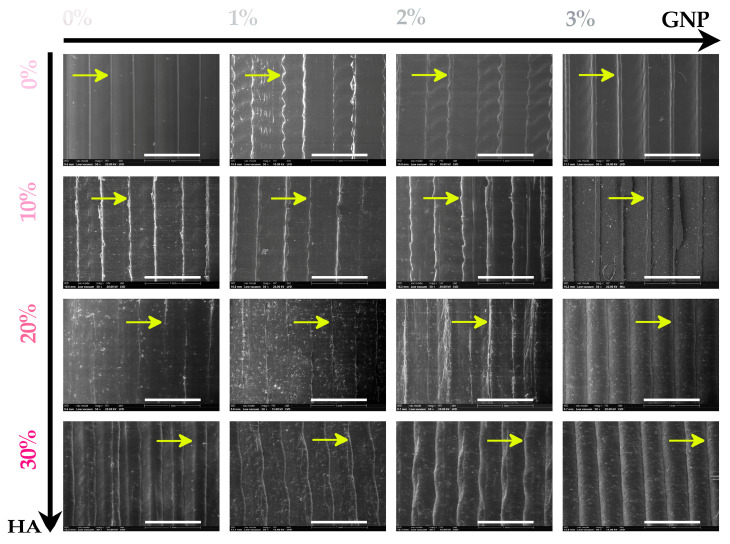
Morphological evaluation on the outer surface of the 3D-printed products prepared with PLA/HA (0–30 wt.%)/GNP (0–3 wt.%). The yellow arrows are indicative of the boundaries of the printed lines. Scale bar: 1 mm.

The morphological features of the 3D-printed products are displayed in Figure 1. The assessment was carried out on the top-view surface in order to disclose the influence of the GNP addition upon the uniform dispersion degree of the HA component into the PLA matrix and their conjoined influence upon the overall integrity and homogeneity of the 3D printed lines.

For all printed products, on each printing line, a uniform dispersion and the embedding of the micrometric HA particles into the polymeric matrix was outlined, with no formation of aggregates. Although possessing slight differences, the micrographs portrayed a sparse emergence and scattering of micrometric bumps/protuberances once the HA ratio increased, compared to the pure PLA sample. This phenomenon was expected given the similar effect of the ceramic particles on the surface features of the composite filaments [31]. Along with the increment of the ceramic component ratio, the fragility/stiffness of the composite materials was identified, in our previous studies, in incompatible ranges with the bone biomedical applications. Thus, the 40 to 50 wt.% HA were excluded from further investigations and from the materials and products development flow.

However, considering the favourable currently reported outcomes, the filaments’ constant diameter and surface–volume homogeneity were reflected into a corresponding homogenous and facile printing of the products, with alleviated material clogging in the printer nozzle. In this regard, the top-view aspect of all layers revealed no detectable interruptions, pores or voids on the surface or at the interface of two printed lines. Yet, at the interface/fusing area, slight deviations from the printing linearity were delineated once the GNP was incorporated (1–2 wt.%). At 2 wt.% GNP mixed with either 20 or 30 wt.% HA, the bulging/cambering effect occurred only for the fusing areas and was preserved along the printed lines. This aftereffect was further enhanced by an increase in the GNP amount in relation to the 20–30 wt.% HA ratios and homogenously propagated across the printed lines, suggesting an improved material fluidity on the GNP’s account.

### 3.2. In Vitro Osteoblasts Response

#### 3.2.1. Cellular Viability and Proliferation Potential

**Figure 2:**
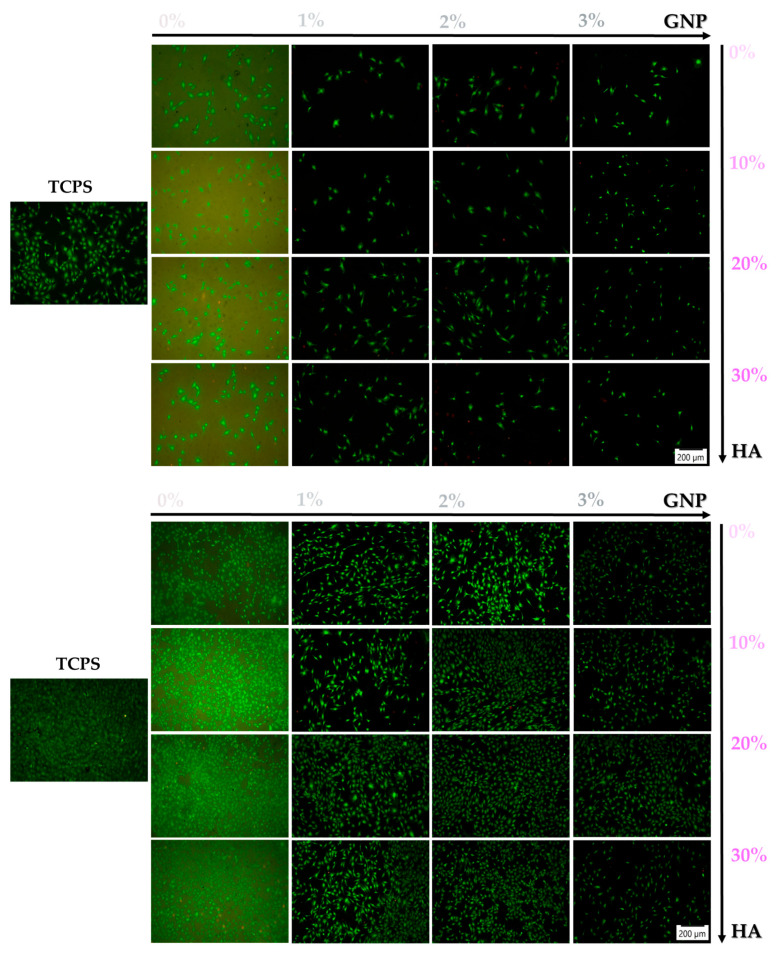
Viability of MC3T3-E1 cells grown in direct contact with the surface of the analysed samples after 24 h and 96 h of culture through the live and dead assay which allows the distinction between viable (green fluorescence) and dead (red fluorescence) cells. Scale bar represents 200 μm.

The short- and long-term survival of the pre-osteoblasts seeded directly onto the surface of the newly developed 3D-printed composites was investigated at 24 h and 96 h post seeding, and the representative fluorescence images are presented in Figure 2. The Live/Dead assay revealed a strong positive ratio between living and dead (red fluorescence) cells, with the predominance of green-stained living cells on all of the analysed samples, thus demonstrating the ability of the novel 3D-printed composites to sustain cellular survival, but with visible differences between samples, and more importantly, when compared to the TCPS control substrate that, according to our expectations and the literature data [37], exhibited the highest number of adherent cells. Moreover, it was reported that physico-chemical features of the TCPS surface turned the substratum into a standard for cell culturing, with a higher and faster proliferative rate in comparison to other implantable biomaterials (e.g., titanium (Ti), polydimethylsiloxane (PDMS), HA, etc.) [38,39,40]. What is noteworthy is that a prolonged culture time led to an increase in cell density over the culture period, suggesting the capacity of the analysed culture substrate to support cell proliferation.

**Figure 3:**
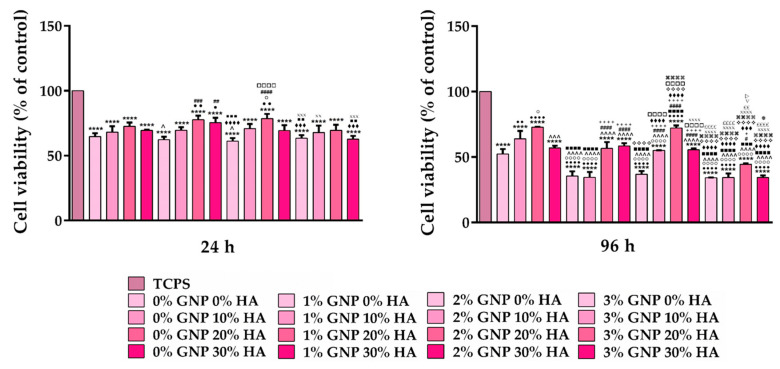
CCK-8 assay highlighting the relative viability vs. the TCPS control sample of the MC3T3-E1 pre-osteoblasts grown in direct contact with the analysed materials for 24 h and 96 h. Data analysis was based on mean ± SD, and the results are expressed as % of control (*n* = 3; **** *p* < 0.0001 vs. TCPS; ●●●● *p* < 0.0001, ●● *p* < 0.01, ● *p* < 0.05 vs. 0% GNP 0% HA; ○○○○ *p* < 0.0001, ○ *p* < 0.05 vs. 0% GNP 10% HA; ^^^^ *p* < 0.0001,^^^ *p* < 0.001, ^ *p* < 0.05 vs. 0% GNP 20% HA; ■■■■ *p* < 0.0001, ■■■ *p* < 0.001, ■■ *p* < 0.01 vs. 0% GNP 30% HA; #### *p* < 0.0001, ### *p* < 0.001, ## *p* < 0.01, # *p* < 0.05 vs. 1% GNP 0% HA; ++++ *p* < 0.0001, + *p* < 0.05 vs. 1% GNP 10% HA; ♦♦♦♦ *p* < 0.0001, ♦♦♦ *p* < 0.001 vs. 1% GNP 20% HA; ❖❖❖❖ *p* < 0.0001 vs. 1% GNP 30% HA; □□□□ *p* < 0.0001 vs. 2% GNP 0% HA; ⌘⌘⌘⌘ *p* < 0.0001 vs. 2% GNP 10% HA; XXXX *p* < 0.0001, XXX *p* < 0.001, XX *p* < 0.01 vs. 2% GNP 20% HA; ££££ *p* < 0.0001, ££ *p* < 0.01 vs. 2% GNP 30% HA;▽ *p* < 0.05 vs. 3% GNP 0% HA; ▷ *p* < 0.05 vs. 3% GNP 10% HA; 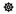 *p* < 0.05 vs. 3% GNP 20% HA).

The lower cell density noticed in the case of the developed materials was also confirmed via the quantitative CCK-8 assay (Figure 3), which showed that the number of metabolically active viable cells found on the surface of the analysed samples suffered an almost 2-fold reduction when compared to the number of cells found on TCPS. Furthermore, the CCK-8 test also revealed significant differences between the survival rates exhibited by the osteoblasts grown onto the surface of the developed materials, a trend observed more prominently at 96 h where the materials containing 0 wt.% and 10 wt.% HA, regardless of the GNP ratio (0–3 wt.%), led to a reduction in the number of viable cells in comparison to the other two remaining samples (20 wt.% and 30 wt.% HA). In addition, this assay showed that the cells grown in contact with the 3 wt.% GNP support, regardless of the HA concentration (0–30 wt.%), displayed statistically significant lower survival rates when compared to the ones cultured onto the surface of the other analysed substrates with lower GNP ratios. These results suggest that the GNP concentration can have a positive influence on the cells’ survival and proliferation rates, but only up to a certain ratio, which, in our case, was 3 wt.%. According to data reported in the literature, the addition of GNP to the biomaterial’s composition can lead to an increased cytotoxicity due to an overly activated apoptotic process [41,42], to positive effects due the molecular interactions established through the GNP groups, which can generate effective pre-concentration platforms for an accelerated cell growth and differentiation [43], or to no effect what so ever [44]. It is worth mentioning that the lower proliferation rates recorded at 96 h do not reflect the supports’ ability to generate cytotoxicity but indicate a reduced proliferative process in comparison to the TCPS control sample. Similar results were reported by Wurn et al., in their study [45], where the osteoblasts grown in contact with PLA discs manufactured through the FDM technique exhibited a significant reduction in their proliferation potential when compared to the TCPS substrate. Moreover, the cell culture polystyrene substrates are usually optimised for a better in vitro cell proliferation, thus leading to the best results as seen within this experiment. Furthermore, this hypothesis is also corroborated by the live and dead results, where no dead red stained cells were observed on the surface of the analysed supports. Overall, the obtained results indicate that the newly developed materials display good in vitro biocompatibility and can sustain cell survival/proliferation to a certain degree. 

#### 3.2.2. Cellular Attachment and Cytoskeleton Organisation 

**Figure 4:**
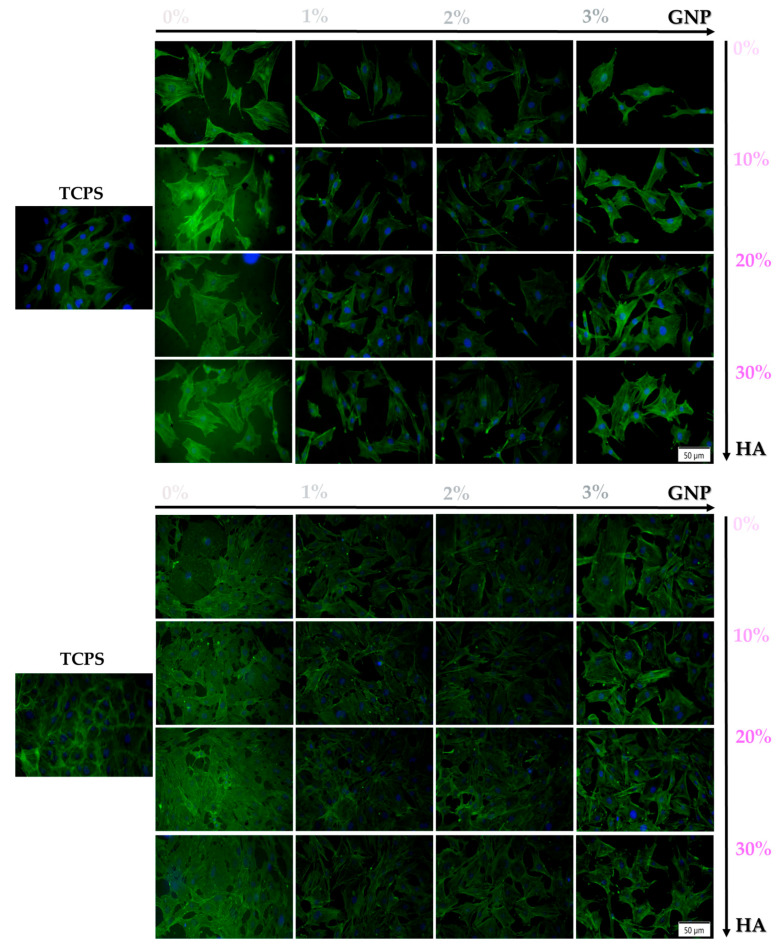
Representative fluorescence images of the morphological characteristics exhibited by the MC3T3-E1 cells grown onto the surface of the tested supports after 24 h and 96 h of culture (actin cytoskeleton—green fluorescence; nuclei—blue fluorescence). Scale bar represents 50 µm.

Since cytoskeleton organisation plays a key role in cell attachment and spreading, in order to confirm the ability of the newly developed 3D-printed supports to sustain and enhance cell adhesion, phalloidin-conjugated Alexa Flour 488 staining was used to highlight the actin filaments at 24 h and 96 h post seeding. The fluorescence microscopic analysis revealed distinctive actin arrangements on all of the analysed surfaces when compared to the TCPS control, where the cells adopted a typical osteoblast morphology and displayed normal organisation of their actin filaments (Figure 4). Thus, after 24 h of culture, the pre-osteoblasts grown onto the surface of the tested samples exhibited a rather elongated morphology with well-pronounced actin fibres and a smaller area of spreading. Moreover, at both experimental time points, regardless of the HA ratio (0–30 wt.%), the 3 wt.% GNP sample presented a lower cellular density when compared to the other composite samples. However, despite the significant reduction in the contact area between cells and surface, the proliferation process was not hindered, and at 96 h, an increase in cell density could be observed for all of the analysed samples. 

Altogether, the fluorescence images indicated a dependency of the cellular adhesion process on the GNP ratio, with the cells grown onto the surface of 3 wt.% GNP materials displaying a distinct behaviour in comparison to the ones grown onto the surface of the other GNP-based analysed samples. 

#### 3.2.3. Osteogenic Differentiation Capacity

**Figure 5:**
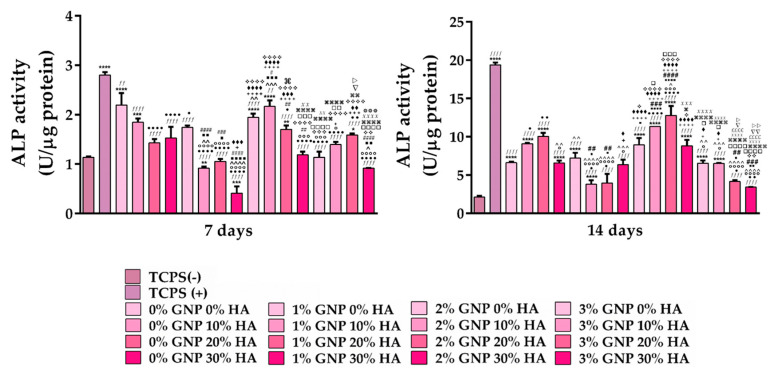
Intracellular ALP activity exhibited by the MC3T3-E1 cells grown directly onto the surface of the analysed supports after 7 and 14 days of culture. Data analysis was based on mean ± SD (n = 3; **** *p* < 0.0001, *** *p* < 0.001, ** *p* < 0.01, * *p* < 0.05 vs. TCPS (-); *ffff p* < 0.0001, *ff* < 0.01 vs. TCPS (+); ●●●● *p* < 0.0001, ●● *p* < 0.01, ● *p* < 0.05 vs. 0% GNP 0% HA; ○○○○ *p* < 0.0001, ○○○ *p* < 0.001, ○ *p* < 0.05 vs. 0% GNP 10% HA; ^^^^ *p* < 0.0001, ^^^ *p* < 0.001, ^^ *p* < 0.01, ^ *p* < 0.05 vs. 0% GNP 20% HA; ■■■■ *p* < 0.0001, ■■■ *p* < 0.001, ■■ *p* < 0.01, ■ *p* < 0.05 vs. 0% GNP 30% HA; #### *p* < 0.0001, ### *p* < 0.001, ## *p* < 0.01, # *p* < 0.05 vs. 1% GNP 0% HA; ++++ *p* < 0.0001, +++ *p* < 0.001, + *p* < 0.05 vs. 1% GNP 10% HA; ♦♦♦♦ *p* < 0.0001, ♦♦♦ *p* < 0.001, ♦♦ *p* < 0.01, ♦ *p* < 0.05 vs. 1% GNP 20% HA; ❖❖❖❖ *p* < 0.0001, ❖❖❖ *p* < 0.001, ❖❖ *p* < 0.01,❖ *p* < 0.05 vs. 1% GNP 30% HA; □□□□ *p* < 0.0001, □□□ *p* < 0.001, □□ *p* < 0.01, □ *p* < 0.05 vs. 2% GNP 0% HA; ⌘⌘⌘⌘ *p* < 0.0001, ⌘⌘ *p* < 0.01, ⌘ *p* < 0.05 vs. 2% GNP 10% HA; XXXX *p* < 0.0001, XXX *p* < 0.001, XX *p* < 0.01 vs. 2% GNP 20% HA; *££££ p* < 0.0001 vs. 2% GNP 30% HA; ▽▽ *p* < 0.01, ▽ *p* < 0.05 vs. 3% GNP 0% HA; ▷▷ *p* < 0.01, ▷ *p* < 0.05 vs. 3% GNP 10% HA; 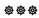 *p* < 0.001 vs. 3% GNP 20% HA). The TCPS (-) and TCPS (+) notations denote the negative and positive controls for pre-osteoblast differentiation, respectively.

Apart from being biocompatible, another desirable feature of biomaterials used as medical devices for bone tissue regeneration is the ability to promote new bone formation by providing an osteoconductive matrix for host osteogenic cells [46], a characteristic which, in turn, would lead to a minimal implant integration period and a reduced risk of implant failure. However, this aspect can only be accomplished if the biomaterial is capable of stimulating and sustaining bone matrix maturation and mineralisation [47]. As an important event in the osteoblasts’ differentiation process, the extracellular matrix formation represents a functional endpoint, which can be easily identified through the intracellular ALP quantification, collagen synthesis and calcium deposition [48]. As an early marker of osteoblast phenotypes, ALP is more than often associated with the progressive osteogenic differentiation process, being up-regulated in its early state and down-regulated during the rest of the differentiation process [49]. In the present study, the ALP activity of the MC3T3-E1 cells seeded directly onto the surface of the newly developed materials was assayed at 7 and 14 days of cultivation, and the obtained results are presented in Figure 5. As seen in the graphic, at 7 days post seeding, the 0 wt.% and 10 wt.% HA supports, regardless of the GNP ratio (0–3 wt.%), showed a considerable increase in the ALP activity in comparison to the 20 wt.% and 30 wt.% HA samples, respectively. However, at 14 days, this trend was no longer observed, and the highest ALP activity was exhibited by the 20 wt.% and 30 wt.% HA samples with a 2 wt.% GNP ratio. However, these results are not surprising, considering the fact that HA can promote osteoblast differentiation. Therefore, higher amounts of HA will generally lead to an enhanced ALP activity. It is worth mentioning that both at 7 days and 14 days post seeding, the ALP levels recorded by the MC3T3-E1 cells grown onto the surface of the analysed materials were significantly reduced when compared to the plastic control in the presence of the osteogenic agents (TCPS (+)), a phenomenon which was observed to be more pronounced after 14 days of culture. A possible explanation for these low levels of ALP activity could be represented by a faster maturation process of the pre-osteoblast grown in contact with the surface of the newly designed materials into osteocytes, cells reported in the literature as expressing low ALP activity [50]. Similar results were noticed by our group in another study [51], where HA-based coatings led to a reduction in the ALP expression levels at 14 days post seeding in comparison to the control sample (flat Ti). Moreover, Genge et al. [52] observed a direct correlation between the loss in the ALP activity and the accumulation of calcium inside the matrix vesicles, a phenomenon associated with the osteogenic differentiation process. 

**Figure 6:**
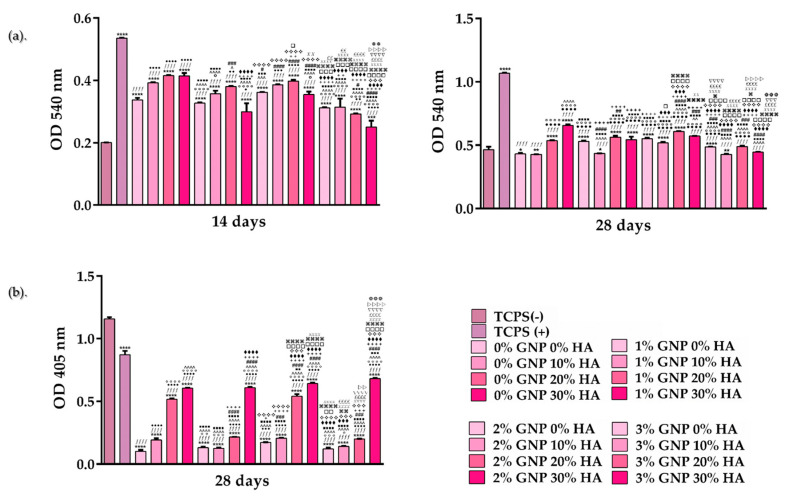
The quantitative analysis of (**a**) collagen synthesis and (**b**) calcium nodules deposition by MC3T3-E1 cells grown directly onto the surface of the analysed samples. Data analysis was based on mean ± SD (n = 3; **** *p* < 0.0001, *** *p* < 0.001, ** *p* < 0.01, * *p* < 0.05 vs. TCPS (-); *ffff p* < 0.0001 vs. TCPS (+) ●●●● *p* < 0.0001, ●●● *p* < 0.001, ●● *p* < 0.01, ● *p* < 0.05 vs. 0% GNP 0% HA; ○○○○ *p* < 0.0001, ○○○ *p* < 0.001, ○○ *p* < 0.01, ○ *p* < 0.05 vs. 0% GNP 10% HA; ^^^^ *p* < 0.0001, ^^^ *p* < 0.001, ^^ *p* < 0.01, ^ *p* < 0.05 vs. 0% GNP 20% HA; ■■■■ *p* < 0.0001, ■■■ *p* < 0.001, ■■ *p* < 0.01 vs. 0% GNP 30% HA; #### *p* < 0.0001, ### *p* < 0.001, ## *p* < 0.01, # *p* < 0.05 vs. 1% GNP 0% HA; ++++ *p* < 0.0001, +++ *p* < 0.001, ++ *p* < 0.01, + *p* < 0.05 vs. 1% GNP 10% HA; ♦♦♦♦ *p* < 0.0001, ♦♦♦ *p* < 0.001 vs. 1% GNP 20% HA; ❖❖❖❖ *p* < 0.0001,❖❖❖ *p* < 0.001 vs. 1% GNP 30% HA; □□□□ *p* < 0.0001, □□□ *p* < 0.001, □□ *p* < 0.01, □ *p* < 0.05 vs. 2% GNP 0% HA; ⌘⌘⌘⌘ *p* < 0.0001, ⌘⌘ *p* < 0.01, ⌘ *p* < 0.05 vs. 2% GNP 10% HA; XXXX *p* < 0.0001, XX *p* < 0.01 vs. 2% GNP 20% HA; *££££ p* < 0.0001, *££ p* < 0.01 vs. 2% GNP 30% HA; ▽▽▽▽ *p* < 0.0001, ▽▽▽ *p* < 0.001 vs. 3% GNP 0% HA; ▷▷▷▷ *p* < 0.0001, ▷▷ *p* < 0.01 vs. 3% GNP 10% HA; 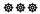 *p* < 0.001, 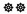 *p* < 0.01 vs. 3% GNP 20% HA). The TCPS (-) and TCPS (+) notations denote the negative and positive controls for pre-osteoblast differentiation, respectively.

In the next step, in order to investigate further osteogenic differentiation, Sirius red and Alizarin red staining were used to highlight collagen synthesis and calcium nodule deposition, respectively. As shown in Figure 6a, after 14 days of culture, the cells seeded onto the 30 wt.% HA sample, regardless of the GNP ratio, exhibited the lowest OD values when compared to the control cells (TCPS (+)) and the cells grown onto the surface of the other GNP-containing analysed substrates. However, at 28 days post seeding, similar to the ALP results, the 20 wt.% and 30 wt.% HA samples were capable of promoting an enhanced collagen synthesis, regardless of the GNP concentrations (0–3 wt.%). Interestingly, the calcium nodule deposition assay revealed significant differences between the analysed samples, with the 30% (regardless of the GNP ratio) and 20 wt.% HA supports (only for the 0 wt.% and 2 wt.% GNP) exhibiting the highest OD values (Figure 6b). This phenomenon could be attributed to the combined action of higher HA concentrations and the tendency of graphene to absorb osteoinductive agents like dexamethasone and β-glycerophosphate [43]. Moreover, data reported in the literature allude to the idea that longer periods of incubation in culture media can led to surface roughness annihilation through an increase in the surface’s ability to adsorb proteins, a phenomenon which, in turn, will lead to enhanced cell attachment and proliferation rates [53].

### 3.3. In Vitro Macrophage Response 

#### 3.3.1. Cellular Survival and Proliferation Potential

**Figure 7:**
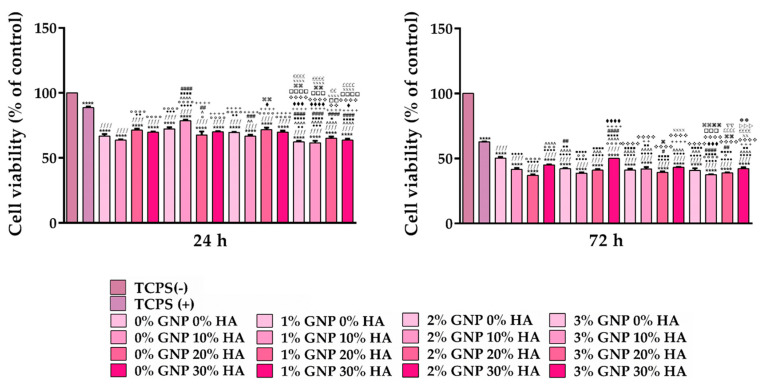
The relative viability vs. the TCPS (-) control sample of the RAW 264.7 macrophages grown in direct contact with the analysed materials, as assessed using the CCK-8 test at 24 h and 72 h post seeding (under proinflammatory stimulation with 100 ng/mL LPS, with the exception of the TCPS (-) sample). Data analysis was based on mean ± SD, and the results are expressed as % of the TCPS (-) control (n = 3, **** *p* < 0.0001 vs. TCPS (-); *ffff p* < 0.0001 vs. TCPS (+); ●●●● *p* < 0.0001, ●●● *p* < 0.001, ●● *p* < 0.01 vs. 0% GNP 0% HA; ○○○○ *p* < 0.0001, ○○○ *p* < 0.001, ○○ *p* < 0.01, ○ *p* < 0.05 vs. 0% GNP 10% HA; ^^^^ *p* < 0.0001, ^^^ *p* < 0.001, ^^ *p* < 0.01, ^ *p* < 0.05 vs. 0% GNP 20% HA; ■■■■ *p* < 0.0001, ■■ *p* < 0.01, ■ *p* < 0.05 vs. 0% GNP 30% HA; #### *p* < 0.0001, ### *p* < 0.001, ## *p* < 0.01, # *p* < 0.05 vs. 1% GNP 0% HA; ++++ *p* < 0.0001, +++ *p* < 0.001, ++ *p* < 0.01 vs. 1% GNP 0% HA; ♦♦♦♦ *p* < 0.0001, ♦♦♦ *p* < 0.001, ♦ *p* < 0.05 vs. 1% GNP 20% HA; ❖❖❖❖ *p* < 0.0001, ❖❖ *p* < 0.01 vs. 1% GNP 30% HA; □□□□ *p* < 0.0001, □□□ *p* < 0.001, □□ *p* < 0.01 vs. 2% GNP 0% HA; ⌘⌘⌘⌘ *p* < 0.0001, ⌘⌘ *p* < 0.01, ⌘ *p* < 0.05 vs. 2% GNP 10% HA; XXXX *p* < 0.0001, XX < 0.01 vs 2% GNP 20 % HA; ££££ *p* < 0.0001, ££ *p* < 0.01 vs. 2% GNP 30% HA; ▽▽ *p* < 0.01 vs. 3% GNP 0% HA; ▷▷▷ *p* < 0.001 vs. 3% GNP 10% HA; 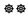 *p* < 0.01 vs. 3% GNP 20% HA). The TCPS (-) and TCPS (+) notations denote the negative and positive controls for inflammation, respectively.

Taking into consideration that the immune response elicited by a biomaterial is heavily dependent on its ability to support cell viability and/or proliferation, in the next step, the viability of RAW 264.7 cells at 24 h and 72 h post seeding was investigated via the CCK-8 test that quantifies the number of viable cells, and the obtained results are presented in Figure 7. As shown in the graphic, at both experimental timepoints, the viability of the macrophages grown in direct contact with the tested samples was significantly lower when compared to the cells cultured in the standard medium without the pro-inflammatory stimulus (TCPS (-) control), a trend observed to be more significant at 72 h post seeding. However, despite the apparent reduction in the number of viable cells, the obtained results do not indicate a potential cytotoxic effect of the investigated composite materials but a reduced cell proliferation potential which could be attributed to the inhibitory effect exerted by the LPS pro-inflammatory stimulus [54]. Moreover, it should be noted that even the TCPS (+) sample, meaning the positive control for inflammation, recorded a reduction in the number of viable cells, which was more obvious after 72 h of culture. In addition, both at 24 h and 72 h, the viability of cells seeded directly onto the surface of the analysed samples was statistically different, with the 20 wt.% and 30 wt.% HA samples exhibiting the highest values. 

#### 3.3.2. Cellular Morphology 

**Figure 8:**
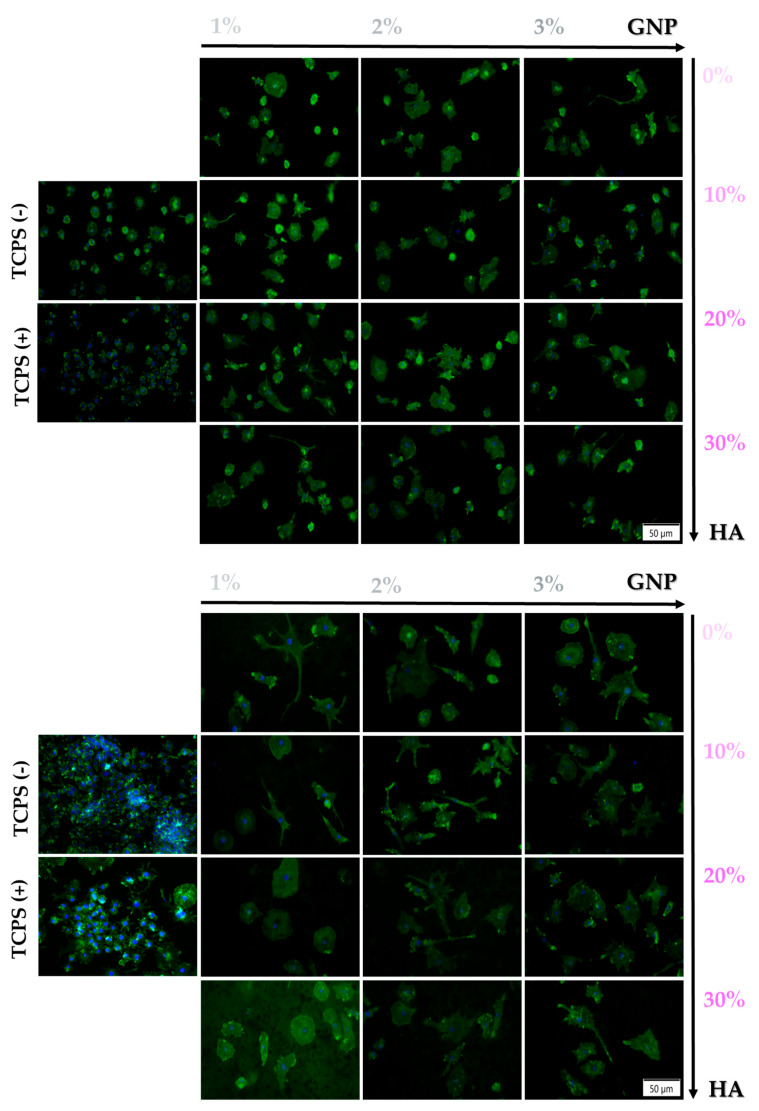
Representative fluorescence images of the RAW 264.7 cells grown for 24 h and 72 h onto the surface of the tested supports, under treatment with LPS (100 ng mL^−1^), with the exception of the TCPS (-) sample (actin cytoskeleton—green fluorescence and nuclei—blue fluorescence). Scale bar represents 50 µm. The TCPS (-) and TCPS (+) notations denote the negative and positive controls for inflammation, respectively. Note: due to its composition and huge number of cells, the 0 wt.% GNP support was impossible to observe under fluorescence microscopy, due to the fact that the PLA-HA matrix absorbed the stain which made the cells undistinguishable from the substrate.

Given the fact that the macrophages are highly versatile cells that can adapt to the microenvironment and acquire specific phenotypes characterised by various morphologies and implicit functions, the morphological features of the RAW 264.7 cells seeded directly onto the surface of the analysed samples were highlighted by staining the actin cytoskeleton with phalloidin conjugated with Alexa Flour 488. Moreover, it is worth mentioning that the macrophages’ structural organisation and architectural characteristics do not only reflect the cells’ ability to adhere and spread on a surface, but also it reveals the differentiation behaviour of those cells [55]. Figure 8 reveals that in the absence of the pro-inflammatory stimulus (TCPS (-)), the cells exhibit normal morphological features for unstimulated macrophages with a small round body, while when stimulated with LPS, the RAW 264.7 macrophages display a higher degree of spreading combined with an altered morphology characterised by an enlarged rounded body shape with discrete cytoplasmic extensions, which can only be associated with an activated pro-inflammatory and migratory M1 phenotype. Moreover, the LPS-treated cells present cytoplasmic dot-like actin structures known as podosomes, which in the literature are described as close-contact zones with the substratum involved in important cellular processes such as mechanosensing, adhesion and matrix degradation [56]. In addition, in terms of the surface cell population, the fluorescence microscopic observation corroborates the CCK-8 test results evinced in Section 3.3.1. 

#### 3.3.3. Nitric Oxide Level Assessment 

**Figure 9:**
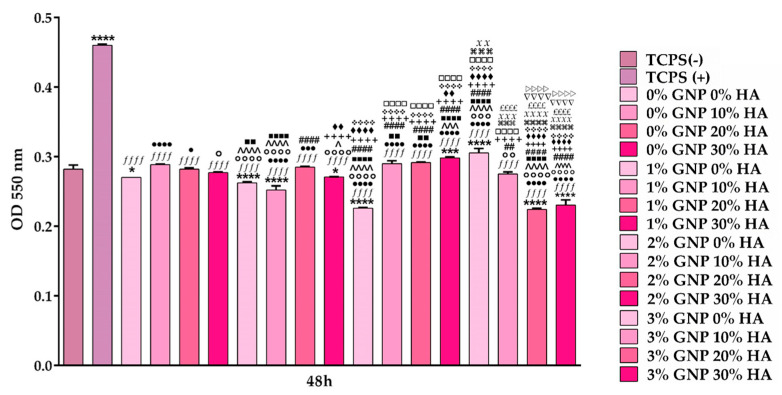
Assessment of the nitrite concentration released in the culture medium by the RAW 264.7 cells seeded directly onto the surface of the tested materials after 48 h in culture (Griess reaction), under pro-inflammatory conditions (100 ng/mL LPS), with the exception of the TCPS (-) sample. Data are presented as mean ± SD (n = 3; **** *p* < 0.0001, *** *p* < 0.001, * *p* < 0.05 vs. TCPS (-); *ffff p* < 0.0001 vs. TCPS (+); ●●●● *p* < 0.0001, ●●● *p* < 0.001, ● *p* < 0.05 vs. 0% GNP 0% HA; ○○○○ *p* < 0.0001, ○○○ *p* < 0.001, ○○ *p* < 0.01, ○ *p* < 0.05 vs. 0% GNP 10% HA; ^^^^ *p* < 0.0001, ^^^ *p* < 0.001, ^ *p* < 0.05 vs. 0% GNP 20% HA; ■■■■ *p* < 0.0001, ■■ *p* < 0.01 vs. 0% GNP 30% HA; #### *p* < 0.0001, ## *p* < 0.01 vs. 1% GNP 0% HA; ++++ *p* < 0.0001 vs. 1% GNP 10% HA; ♦♦♦♦ *p* < 0.0001, ♦♦ *p* < 0.01 vs. 1% GNP 20% HA; ❖❖❖❖ *p* < 0.0001 vs. 1% GNP 30% HA; □□□□ *p* < 0.0001 vs. 2% GNP 0% HA; ⌘⌘⌘⌘ *p* < 0.0001, ⌘⌘⌘ *p* < 0.001 vs. 2% GNP 10% HA; XXXX *p* < 0.0001, XXX *p* < 0.001, XX *p* < 0.01 vs. 2% GNP 20% HA; ££££ *p* < 0.0001 vs. 2% GNP 30% HA; ▽▽▽▽ *p* < 0.0001 vs. GNP 3% HA 0%; ▷▷▷▷ *p* < 0.0001 vs. 3% GNP 10% HA). The TCPS (-) and TCPS (+) notations denote the negative and positive controls for inflammation, respectively.

When NO was first introduced to the immunology community, its inflammatory role was defined in very basic terms, and even though the fundamental definition is still widely accepted, during the past few decades, its multiple inflammatory roles have been reported and acknowledged. Thus, nowadays, NO is recognised to be one of the most crucial and versatile molecules involved in the regulation of acute and chronic inflammation, having a dual role both as a pro- and anti-inflammatory agent [57]. Since NO generation is recognised as a specific feature of activated macrophages, the effects of the newly developed 3D-printed composites on the LPS-induced NO production were evaluated by quantifying the concentrations of nitrite accumulated in the culture media after 48 h of culture. From Figure 9, it can be seen that the analysed samples led to a statistically significant decrease in the production of NO in comparison to the positive control for inflammation (TCPS (+)) (*p* < 0.0001). In contrast, the negative control represented by cells grown in standard culture conditions (TCPS (-)) showed either similar or significantly lower nitrite concentrations when compared to the tested samples (*p* < 0.0001; *p* < 0.001; *p* < 0.05). Additionally, across all of the analysed samples, the 0 wt.% and 1 wt.% GNP samples led to significantly lower nitrite concentrations in comparison to the other two samples containing 2 wt.% and 3 wt.% GNP. Moreover, the obtained results could explain the viability rates of macrophages seeded directly onto the surface of the analysed samples, since NO is a key player both in cell viability and apoptotic death regulation [58] through its influence on the cell’s mitochondrial function. However, data reported in the literature suggest that the impact of NO on cell viability is quite variable, mostly depending on the cell type and the amount of NO that is produced. For example, moderate NO levels can exert a protective role, thus inhibiting the apoptotic process, while high concentrations, like those that are characteristic of the inflammatory response, lead to a decrease in the cells’ viability via apoptotic death promotion. 

#### 3.3.4. In Vitro Macrophage Fusion Assay

Among all immune cell types, under certain circumstances (e.g., pathological conditions or reactions towards non-infectious foreign body materials such as implants, prostheses and medical devices), macrophages can fuse and form FBGCs—a subtype of multinucleated giant cells characterised by an irregular body shape and a multitude of nuclei dispersed throughout the cytoplasm [59]. Since macrophages fusion processes and their subsequent FBGC formation represent a hallmark of biomaterial-induced chronic inflammation [60], in the present study, we evaluated the extent of multinuclear FBGC formation after 7 days of culture, both in the absence and presence of the pro-inflammatory agent LPS (100 ng/mL), and the representative fluorescence images are shown in Figure S2 (Supplementary Material). Thus, the microscopic images revealed that the treated RAW 264.7 cells presented altered morphological characteristics, displaying larger bodies with multiple nuclei, while the cells cultured in the standard medium retained their smaller round body shape with one single nucleus. In addition, the extent of the macrophage fusion process was quantified by determining the “multinuclear index” expressed in percentages (%). Thus, after LPS stimulation, it was noticed that by increasing the GNP ratio, the multinuclear index was reduced, and a possible explanation for this phenomenon could be the fact that at high concentrations, e.g., 3 wt.%, the number of cells found on the surface of these materials suffered a slight reduction in comparison to the other supports (with lower GNP ratios), therefore leading to a decreased macrophage fusion process and implicit FBGC formation. Moreover, in the absence of LPS, the values obtained indicated an almost 2-fold reduction in the multinuclear FBGC incidence on almost all of the analysed surfaces, suggesting that the 3D-printed composite substrates are capable of reducing the inflammatory response once implanted into the human body. 

## 4. Conclusions

The fabrication of 3D-printed scaffolds is an emerging multidisciplinary field, especially in the bone tissue regeneration area, mainly due to the technique’s versatility in terms of building complex shapes and custom-designed parts with targeted properties. With this in mind, in the present study, GNP-reinforced PLA/HA composite scaffolds were successfully designed and fabricated through an extrusion-based 3D printing technique (FDM).

- The overall features of the scaffolds were evaluated, and the morphological evaluation (top-view surface) revealed a uniform dispersion and the embedding of the ceramic particles, with no formation of aggregates regardless of the GNP amount.
- The conversion from smooth to slightly rough surfaces was observed at higher HA ratios, leading to the emergence of micrometric protuberances. Once the GNP amount reached the maximum value (3 wt.%) and the HA ratio was in the 20–30 wt.% range, a bulging effect occurred and was preserved along the printed lines, suggesting an improved material fluidity; yet, this aftereffect is not always adequate for a proper cellular response.
- The in vitro biological assessment using two different cell lines, MC3T3-E1 pre-osteoblasts and RAW 264.7 macrophages, showed that all scaffolds, to different degrees, are suitable for cell growth and that the presence of HA in higher ratios (20 wt.% and 30 wt.%) created a positive osteogenic microenvironment capable of stimulating new bone formation.
- Moreover, a desirable inflammatory activity was observed for all of the analysed scaffolds, but in different degrees.

Overall, the obtained data suggest that the newly developed PLA/HA/GNP materials could offer an easy and efficient alternative for designing biomaterials that can provide a supportive osteoimmunomodulatory microenvironment for the regeneration of bone defects, with potential applications in orthopedic surgery. However, despite the promising in vitro results, due to variations resulting from manufacturing and laboratory conditions, a more in-depth exploration of the PLA/HA/GNP supports is absolutely necessary in order to ensure their increased performance in future clinical applications. Thus, a further direction for research inspired by this study could a long-term investigation of the newly developed scaffolds, both in terms of mechanical characteristics and in vivo studies, that could offer additional information, which would validate the materials’ efficacy and safety for biomedical applications. 

## Data Availability

Data are contained within the article.

## Supplementary Materials

The following supporting information can be downloaded at: https://www.mdpi.com/article/10.3390/biomimetics9010055/s1, Figure S1: Scaffold manufacturing—graphical presentation; Figure S2: (**A1**–**A4**) Representative fluorescence images highlighting the formation of multinucleated FBGC both under standard (-LPS) and pro-inflammatory conditions (+LPS, stimulation with 100 ng/ml LPS) (actin cytoskeleton—green fluorescence; nuclei- blue fluorescence). Scale bar represents 50 µm; (**B1**–**B4**) The values of the “multinuclear index” as determined by examining 10–14 microscopic fields for each sample. The TCPS (-) and TCPS (+) notations denote the negative and positive controls for inflammation, respectively.
