# Supplementary material for: In Vitro Studies on 3D-Printed PLA/HA/GNP Structures for Bone Tissue Regeneration
Source: Biomimetics (Basel). 2024 Jan 19;9(1):55. doi: 10.3390/biomimetics9010055 (PMC10813057; doi:10.3390/biomimetics9010055)
Supplement: Supplementary file 1 [file biomimetics-09-00055-s001.zip › biomimetics-2770129-supplementary.pdf]

biomimetics

Supplementary Material

Andreea-Mariana Negrescu [1], Aura-Cătălina Mocanu [2], Florin Miculescu [2], Valentina Mitran [1], Andreea-Elena Constantinescu [2] and Anisoara Cimpean [1,*]

[1] Department of Biochemistry and Molecular Biology, Faculty of Biology, University of Bucharest, 91-95 Splaiul Independentei, 050095 Bucharest, Romania; andreea.ne94@gmail.com (A.-M.N.); valentina.mitran@bio.unibuc.ro (V.M.)
[2] Department of Metallic Materials Science, Physical Metallurgy, National University of Science and Technology Politehnica Bucharest, 313 Splaiul Independentei, J Building, District 6, 060042 Bucharest, Romania; mcn_aura@hotmail.com (A.-C.M.); f_miculescu@yahoo.com (F.M.); andreeaelena01c@gmail.com (A.-E.C.)
* Correspondence: anisoara.cimpean@bio.unibuc.ro; Tel.: +40-21-318-1575 (ext. 106)

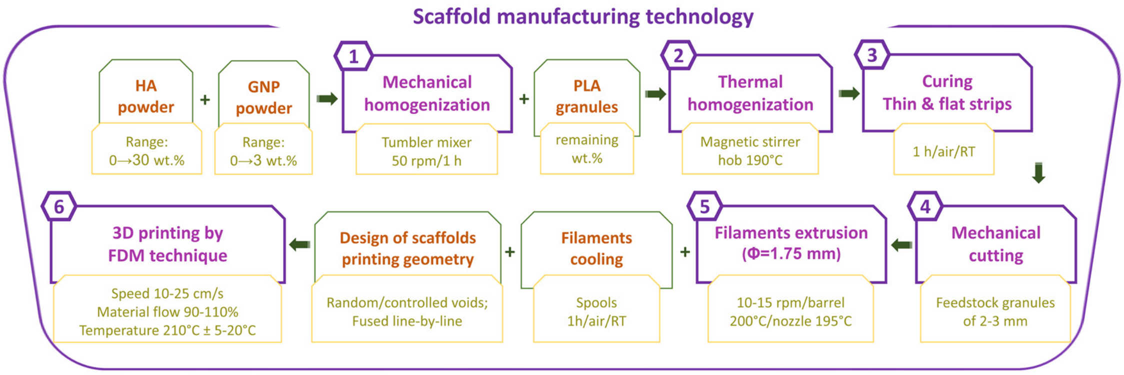

Figure S1. Scaffold manufacturing - graphical presentation.

A1.

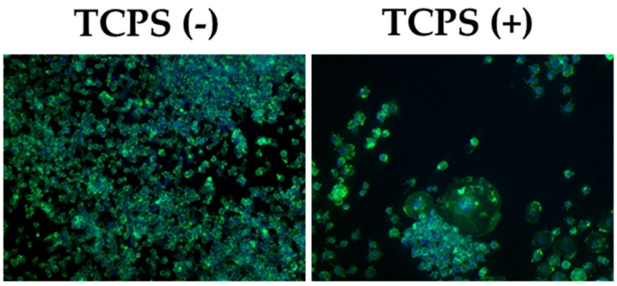

B1.

Sample (-LPS)		Total number of nuclei	Number of nuclei in multinuclear cells	Multinuclear index (%)
0 % GNP	0% HA	26	3	11.5%
	10% HA	85	3	3.5%
	20% HA	63	4	6.3%
	30% HA	38	0	0%
TCPS (-)		324	3	0.9%

Sample (+LPS)		Total number of nuclei	Number of nuclei in multinucleate cells	Multinuclear index (%)
0 % GNP	0% HA	62	0	0%
	10% HA	48	3	6.25%
	20% HA	51	3	5.8%
	30% HA	28	0	0%
TCPS (+)		30	8	26.6%

Note: Due to its composition and huge number of cells, the 0 wt.% % GNP support was impossible to be observed under fluorescence microscopy due to the fact that the PLA-HA matrix absorbed the stain which made the cells undistinguishable from the substrate.

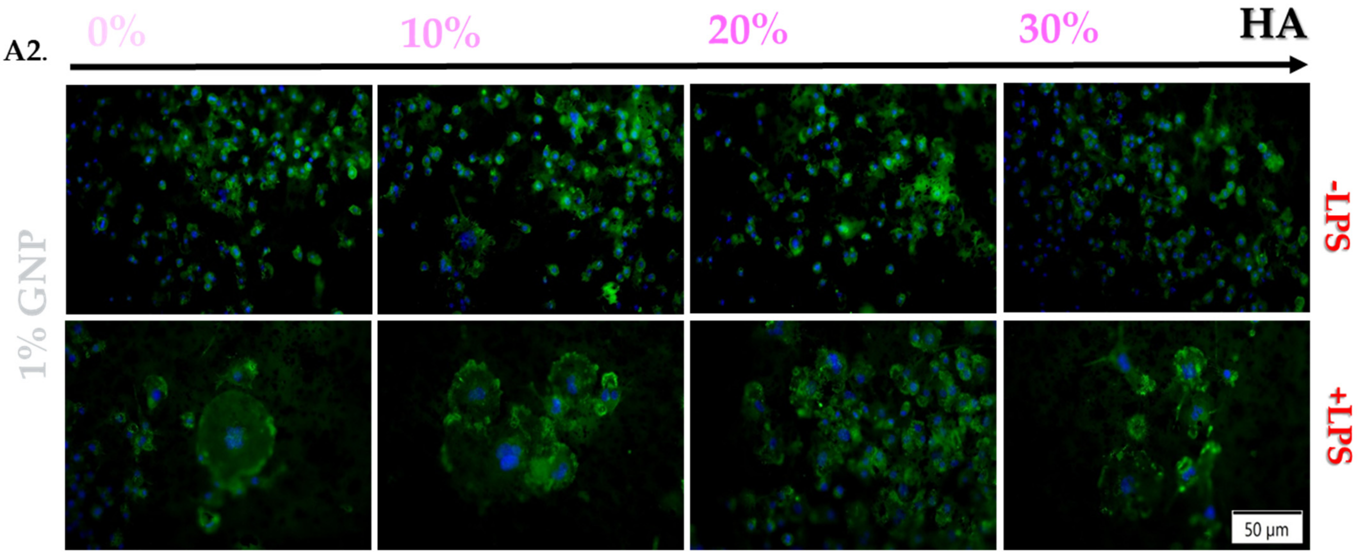

B2.

Sample (-LPS)		Total number of nuclei	Number of nuclei in multinuclear cells	Multinuclear index (%)
1% GNP	0% HA	129	7	5.4%
	10% HA	101	3	3.12%
	20% HA	58	3	5.1%
	30% HA	99	3	3.03%

Sample (+LPS)		Total number of nuclei	Number of nuclei in multinucleate cells	Multinuclear index (%)
1% GNP	0% HA	60	5	8.3%
	10% HA	59	3	5.8%
	20% HA	54	6	11.1%
	30% HA	59	6	10.1%

A3.

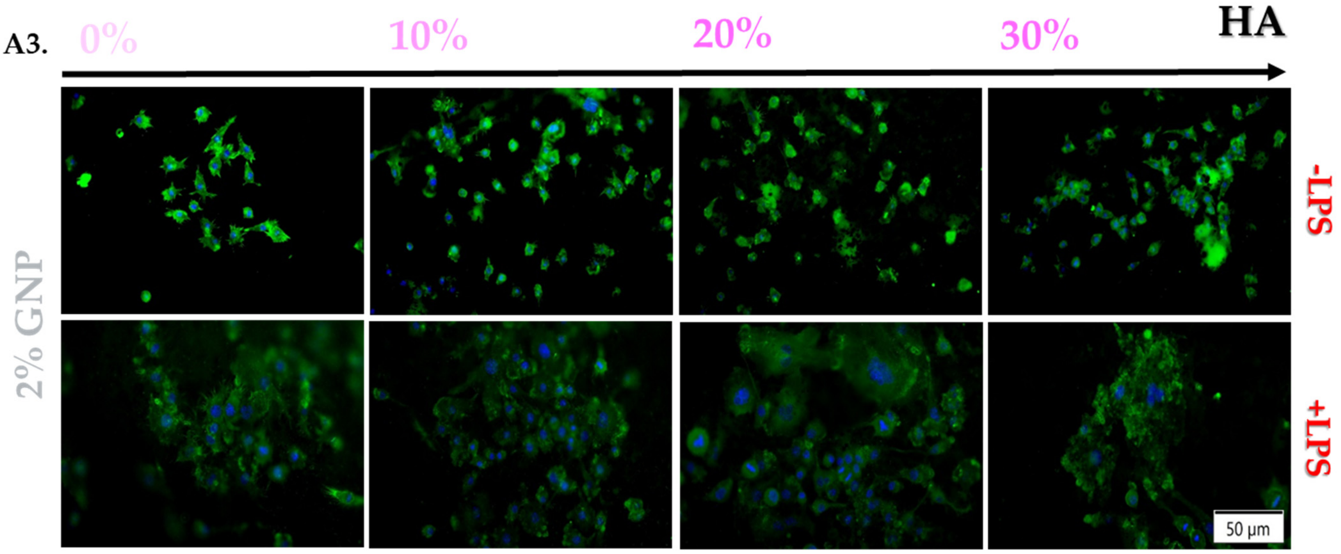

B3.

Sample (-LPS)		Total number of nuclei	Number of nuclei in multinuclear cells	Multinuclear index (%)
2% GNP	0% HA	19	0	0%
	10% HA	53	3	5.66%
	20% HA	47	0	0%
	30% HA	63	3	4.76%

Sample (+LPS)		Total number of nuclei	Number of nuclei in multinucleate cells	Multinuclear index (%)
2% GNP	0% HA	38	3	7.8%
	10% HA	36	3	8.3%
	20% HA	74	6	9.6%
	30% HA	27	3	11.1%

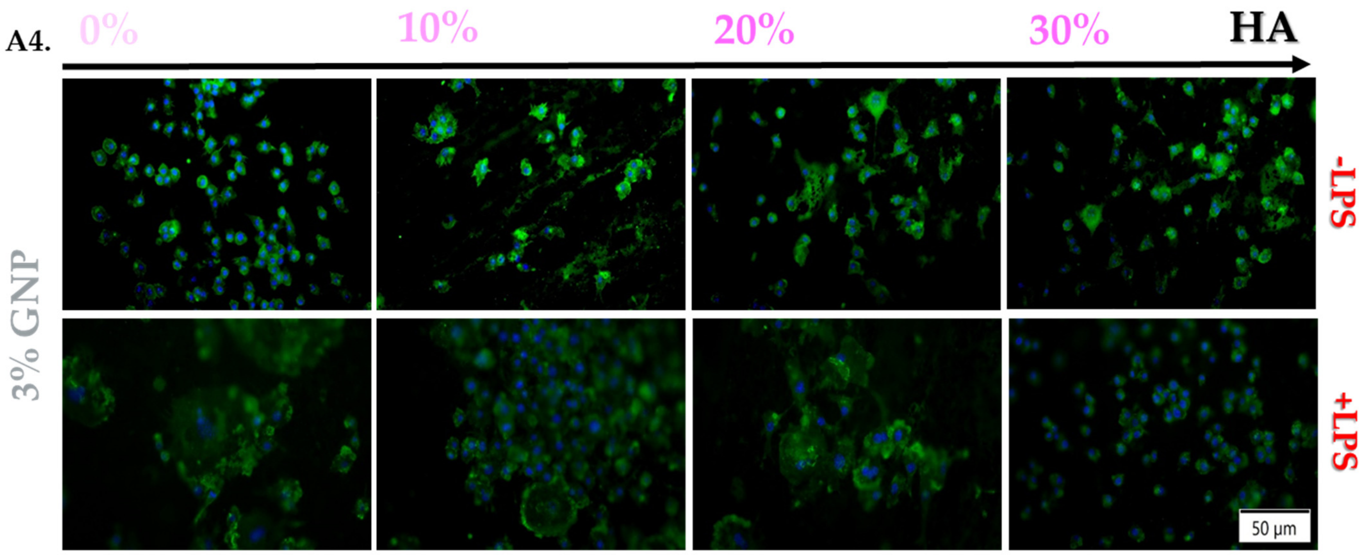

B4.

Sample (-LPS)		Total number of nuclei	Number of nuclei in multinuclear cells	Multinuclear index (%)
3% GNP	0% HA	59	3	5.08%
	10% HA	38	3	7.8%
	20% HA	58	0	0%
	30% HA	75	3	4%

Sample (+LPS)		Total number of nuclei	Number of nuclei in multinucleate cells	Multinuclear index (%)
3% GNP	0% HA	59	3	5.08%
	10% HA	63	3	6.3%
	20% HA	141	3	2.11%
	30% HA	130	3	2.3%

Figure S2 (A1-A4). Representative fluorescence images highlighting the formation of multinucleated FBGC both under standard (-LPS) and pro-inflammatory conditions (+LPS, stimulation with 100 ng/ml LPS) (actin cytoskeleton – green fluorescence; nuclei- blue fluorescence). Scale bar represents 50 µm; **(B1-B4)** The values of the "multinuclear index" as determined by examining 10–14 microscopic fields for each sample. The TCPS (-) and TCPS (+) notations denote the negative and positive controls for inflammation, respectively.
